# Circular Permutation of Red Fluorescent Proteins

**DOI:** 10.1371/journal.pone.0020505

**Published:** 2011-05-27

**Authors:** Bo Shui, Qi Wang, Frank Lee, Laura J. Byrnes, Dmitry M. Chudakov, Sergey A. Lukyanov, Holger Sondermann, Michael I. Kotlikoff

**Affiliations:** 1 Department of Biomedical Sciences, College of Veterinary Medicine, Cornell University, Ithaca, New York, United States of America; 2 Department of Molecular Medicine, College of Veterinary Medicine, Cornell University, Ithaca, New York, United States of America; 3 Shemyakin-Ovchinnikov, Institute of Bioorganic Chemistry, Russian Academy of Science, Moscow, Russian Federation; University of Delhi, India

## Abstract

Circular permutation of fluorescent proteins provides a substrate for the design of molecular sensors. Here we describe a systematic exploration of permutation sites for mCherry and mKate using a tandem fusion template approach. Circular permutants retaining more than 60% (mCherry) and 90% (mKate) brightness of the parent molecules are reported, as well as a quantitative evaluation of the fluorescence from neighboring mutations. Truncations of circular permutants indicated essential N- and C- terminal segments and substantial flexibility in the use of these molecules. Structural evaluation of two cp-mKate variants indicated no major conformational changes from the previously reported wild-type structure, and *cis* conformation of the chromophores. Four cp-mKates were identified with over 80% of native fluorescence, providing important new building blocks for sensor and complementation experiments.

## Introduction

Circular permutation of GFP and its variants has markedly expanded the utility of fluorescent proteins (FPs), enabling the development of genetically encoded sensors [1], [2], [3] and facilitating the use of FPs in fluorescence complementation assays [4], [5]. The extension of this technology to red fluorescent proteins, while potentially quite useful, has been limited; bright and thermostable circular permutants or complementary peptides have been difficult to design [1], [2], likely because the local chromophore environment is altered by the rearrangements attempted to date [6]. In an effort to address this limitation, we undertook a systematic effort to produce bright and stable circularly permutated variants of two naturally occurring proteins, mCherry, a monomeric variant of the *Discosoma sp.* coral protein DsRed [7], and mKate, a monomeric variant of the anemone *Entacmaea quadricolor* protein eqFP578 [8], [9]. Both proteins have advantageous tissue imaging properties, including relative brightness and long wavelength emission characteristics; moreover, both are spectrally distinct from GFP and its derivatives, potentially expanding the color palette of genetically encoded sensors or complementation pairs that can be used simultaneously with GFP-based constructs. Here we report the systematic evaluation of circular permutation sites in mCherry and mKate, and the development of highly efficient circularly permutated red fluorescent proteins (cp-RFPs).

## Materials and Methods

### Generation of cp-RFPs from Tandem Fusion Templates

**Figure 1:**
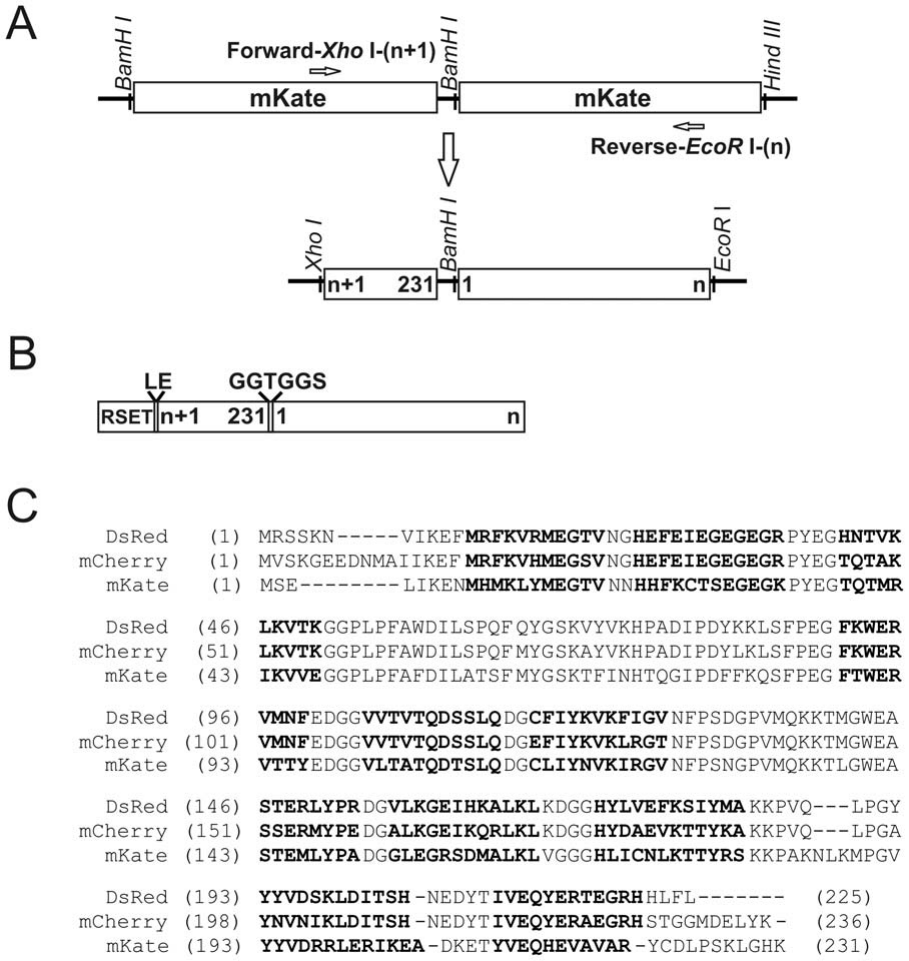
Schematic plasmid and protein sequences of cp-Red Fluorescent Proteins. **A.** Circular permutants were constructed from a tandem fusion template with forward (new N-terminal) and reverse (new C-terminal) primers, producing a full length amplicon beginning and ending at any desired site. Strategy for mKate circular permutation is shown. **B.** Diagram of cp-RFP final proteins with the N-terminal leader peptide from the expression vector pRSET-A; C (n+1 to 231) and N (1 to n) -terminal residues of mKate were joined by a GGTGGS linker and the LE linker between RSET and cp-mKate was encoded by the *Xho*I site. **C.** Amino acid sequences of DsRed from the *Discosoma sp.* coral, DsRed-derived mCherry, and mKate from the sea anemone *Entacmaea quadricolor*. Chromophore forming residues are underlined; beta-strands forming residues are shown in bold.

To efficiently probe multiple permutation sites, we created tandem fusion templates [10] of mKate (pRSET-tdmKate) and mCherry (pRSET-tdmCherry). For the former, the mKate (231 AA) coding sequence was amplified from plasmid TagFP635(Evrogen) using Phusion High-Fidelity DNA Polymerase (Finnzymes) with copy 1, forward (F1) *BamH*I-mK-1F (atca*ggatcc*atgtctgagctgattaaggaga) and reverse (R1) *Hind*III-mK-231R (ctac*aagctt*tcatttgtgccccagtttgctagg) primers (restriction sites intalicized). The amplified product was inserted into the *BamH*I/*Hind*III sites of pRSET-A (Invitrogen) to produce pRSET-mKate. The second copy of the mKate reading frame was amplified from the TagFP635 template with (F2) *BamH*I-mK-1F and (R2) *BamH*I-mK-231R-linker (ctac*ggatcc* gccggtaccgcctttg tgccccagtttgctagg) primers. Note the absence of a stop codon in the reverse primer (R2) and the underlined linker such that the *BamH*I site provides the residues GS in the linker peptide GGTGGS. The PCR product was inserted into the *BamH*I site of pRSET-mKate and proper orientation was determined by PCR and DNA sequencing. The final plasmid (pRSET-tdmKate) contains two tandem mKate coding sequences separated by the coding sequence for linker peptide GGTGGS, in a contiguous reading frame (**Fig. 1A**). A similar strategy was used to construct pRSET-tdmCherry using the mCherry (236 AA) coding sequence from pRSET-B mCherry [7], primers (F1) *BamHI*-mC-1F (ctac*ggatcc*atggtgagcaagggcgaggagga), (R1) *Hind*III-mC-236R (ctac*aagctt*tcacttgtacagctcgtccat), (F2) *BamH*I-mC-1F and (R2) *BamH*I-mC-236R-linker (ctac*ggatcc* gccggtaccgcccttgtacagctcgtcca). The tandem fusion templates were used as PCR templates to generate cp-RFPs by systematically varying the N- and C-terminal primers. To construct cp-mKate^156-155^, the *Xho*I site was replaced with the *Nhe*I in the forward primer and the amplified fragment was cloned into the *Nhe*I and *Eco*RI sites of pRSET-A to prevent recreation of cp-mKate^154-155^ variant (Fig. 1B,C). Truncated cp-mKate variants were constructed from the tandem fusion mKate template using a similar PCR strategy.

### Screening of cp-RFPs in *E.coli*

*E.coli* BL21 Star (DE3) pLysS (Invitrogen) cells were transformed with either pREST-cp-mCherry or pRSET-cp-mKate variant plasmids and plated on LB agar plates supplemented with 100 µg/mL ampicillin. After incubation at 37°C for 24 h, colonies were screened for red fluorescence using a widefield macroimaging system (OV100, Olympus, Japan; 545 nm excitation/570–625 nm emission). Colonies were rescreened after 1–7 days at 2–8°C. Images were analyzed for brightness using Image J and normalized to mCherry or mKate –transformed colonies; vector control colonies were used as the zero fluorescence background.

### Protein Expression and Purification

Overnight seed cultures of transformed BL21 Star (DE3) pLysS cells were used to inoculate (1∶40 dilution) 500 mL Terrific Broth containing 100 µg/mL ampicillin. The cultures were incubated at 37°C (250 rpm) until OD_600_ ∼1.0; 0.5 mM IPTG was added and cultures incubated for 16 h at 20°C (250 rpm). Cells were collected by centrifugation at 6000×g for 10 minutes at 4°C and resuspended in 20 mM HEPES (pH7.4) with 350 mM NaCl and 0.1% TritonX-100. Cultures were sonicated and cell debris removed by centrifugation at 20,000×g for 30 minutes at 4°C. The cleared lysate was mixed with 3 ml of Profinity IMAC Ni-Charged Resins (Bio-Rad) and incubated on a rocker platform for 5 minutes, the resin poured onto a 0.8×4 cm chromatography column (Bio-Rad), washed 3 times with 20 mM HEPES (pH 7.4) buffer containing 350 mM NaCl and 0.1% TritonX-100, and 3 times with 20 mM HEPES (pH 7.4) containing 350 mM NaCl; each wash was followed by low speed centrifugation at 800×g for 30 seconds. Proteins were eluted with 20 mM HEPES (pH 7.8) containing 350 mM NaCl and 300 mM imidazole buffer and the pooled protein fractions desalted and concentrated using an Amicon Ultra-15 device (Millipore) with 20 mM HEPES (pH 7.4), refilling 2–3 times. Protein purity was checked by SDS-PAGE and the concentration was measured (Pierce BCA Protein Assay).

### Spectroscopy of cp-RFPs

The spectral properties of RSET-tagged proteins in 20 mM HEPES (pH 7.4) were measured at 20°C. Absorption spectra were acquired on a DU 730 UV/visible spectrophotometer (Beckman Coulter), and fluorescence spectra on a FluoroMax-3 spectrofluorimeter (Jobin Yvon Horiba). Extinction coefficients of cp-mCherry proteins were measured using the alkali denaturation method with 0.1 M NaOH [11], [12]. Quantum yield (Φ) for cp-mCherrys and cp-mKates was measured using the parent molecules (mCherry: Φ = 0.22; mKate: Φ = 0.33) as the reference standards [13]. The fluorescence of 5 µM protein solutions in 20 mM HEPES (pH 7.4) was measured using a Synergy 2 Multi-Mode Microplate Reader (Biotek; excitation 575/15, emission 620/15).

### Crystallization and Structure Determination

For crystallization the coding region of cp-mKate^154-153^ and cp-mKate^168-167^ was amplified and cloned into a modified pET28a expression plasmid (Novagen) yielding an N-terminal hexahistidine SUMO fusion protein, with the tagged moiety cleavable with the protease Ulp-1 from *S. cervisiae*.

*E.coli* BL21 (DE3) cells (Novagen) were transformed by cp-mKate plasmids. Protein expression and purification were performed according to protocols described previously [6]. Crystals were obtained by hanging drop vapor diffusion by mixing equal volumes of protein (∼35 mg/ml) and reservoir solution followed by incubation at 20°C. Crystals were obtained with the reservoir solution containing 0.1 M Tris-HCl pH 7.4, 0.2 M MgCl_2_ and 18% PEG3350 (for cp-mKate^154-153^) or 0.1 M Magnesium formate dihydrate and 14% PEG 3350 (for cp-mKate^168-167^). All crystals were cryo-protected using crystallization solutions supplemented with 20% xylitol, frozen in liquid nitrogen, and kept at 100 K during data collection.

Data sets were collected using synchrotron radiation at the Cornell High Energy Synchrotron Source (CHESS, Ithaca). Data reduction was carried out with the software package HKL2000 [14]. Phases were obtained from molecular replacement using the software package PHENIX [15] with the available structure of mKate (1.8 Å, pH 2.0, PDB code: 3BX9) [16] as the search models. Manual refinement in COOT [17] and minimization using PHENIX [15] yielded the final models with good geometry.

## Results

### Circular Permutation of mCherry

**Figure 2:**
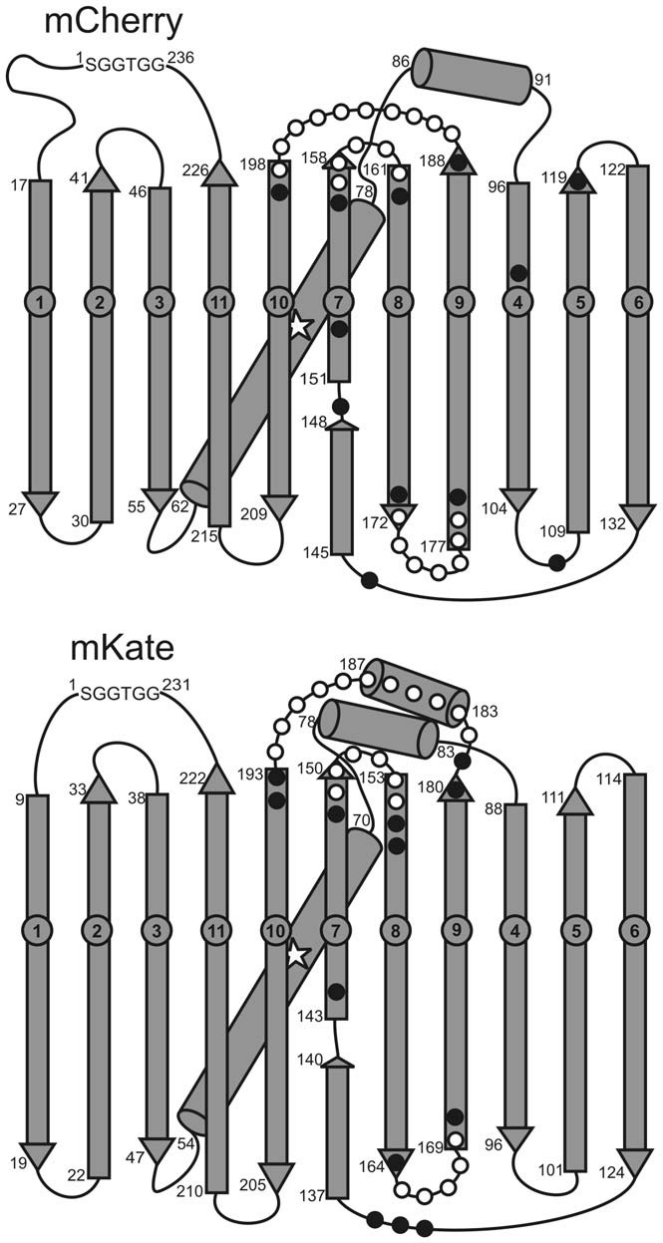
Circular permutation sites scanned in mCherry and mKate. mCherry and mKate were numbered by primary amino acid sequences as shown in **Fig. 1C**. Open circles indicate sites with fluorescence, closed circles sites without fluorescence. Three highly homologous regions of circular permutation tolerant sites in mCherry and mKate: Loop 7–8 region located in the loop between the 7^th^ and 8^th^ β-strands and flanking sites on the β-strands, Loop 8–9 region located in the loop between the 8^th^ and 9^th^ β-strands and flanking sites on the β-strands, and Loop 9–10 region flanked by the 9^th^ and 10^th^ β-strands.

To efficiently scan mCherry for promising circular permutation sites we created multiple cp-mCherry clones by PCR amplification of a tandem-mCherry template (pRSET-tdmCherry, Methods) and directly compared the red fluorescence of bacterial colonies with those transformed by pRSET-mCherry. Initial cp-mCherry constructs targeted the loops of mCherry from the 4^th^ to 10^th^ β-strands (**Fig. 2**). None of the cp-mCherry variant colonies exhibited appreciable fluorescence after overnight incubation at 37°C when compared with colonies transformed with wildtype mCherry, whereas after further incubation for 24 h at either room temperature or 2–8°C, fluorescence was detected in cp-mCherry^158-157^, cp-mCherry^175-174^, and cp-mCherry^193-192^ colonies. Other variant colonies did not exhibit appreciable fluorescence even after 7 days at 2–8°C (data not shown).

Initial screening identified three sites in distinct loops connecting β strands. We probed the flanking sequences at each of these sites by constructing and analyzing additional cp-mCherry variants. The brightest colony (cp-mCherry^194-193^) exhibited only 1% of mCherry after 24 h incubation at 37°C, indicating that the circularly permutated proteins matured slowly in *E.coli*. Based on this observation all screens were scored after 24 h at 37°C and further 72 h at 2–8°C (**Table 1**). Colony fluorescence data indicated that permissive sites were grouped in three loops between 7^th^ and 8^th^ β-strands, 8^th^ and 9^th^ β-strands, 9^th^ and 10^th^ β-strands, and extending to ends of flanking β-strands in each region, respectively (**Fig. 2**). Validation of the bacterial colony fluorescence assay with fluorescence measurements of purified cp-mCherry proteins in 20 mM HEPES (pH 7.4) indicated that cp-mCherry^194-193^ is the brightest variant from all of the screened circular permutations, displaying approximately 90% relative brightness on a chromophore basis and 60.6% protein brightness compared to mCherry (**Table 2**). The difference between these measurements indicates suboptimal protein folding, as discussed below. cp184-mCherry was previously reported to display 18% of native mCherry fluorescence when proteins are expressed in *E.coli*, and 37% of fluorescence when the isolated proteins are compared [18]. Evolution of cp193-mCherry to cp193g7 (corresponding to cp-mCherry^197-196^) by random mutagenesis improved the brightness of this variant, achieving 61% of mCherry brightness on a protein basis [19].

**Table 1:** Screening of circular-permutated mCherry variants in *E.coli*.

Variant *	Colony brightness 24 h at 37°C (% of mCherry)	Colony brightness 72 h at 2–8°C (% of mCherry)
mCherry	100	100
pRSET-A	0	0
cp99-98	0	0
cp107-106	0	0
cp119-118	0	0
cp143-142	0	0
cp149-148^b^	0	0
cp153-152	0	0
**Loop 7–8 region and flanking sites**		
cp156-155	0	0
cp157-156	0	0.7
cp158-157^a^	0	2.8
cp159-158	0.1	17.5
cp160-159	0.1	12.3
cp161-160	0	0.3
cp162-161	0	0
**Loop 8–9 region and flanking sites**		
cp171-170	0	0
cp172-171	0	0.4
cp173-172	0	1.1
cp174-173	0.1	6.4
cp175-174^a^	0.3	19.8
cp176-175	0	3.9
cp177-176	0	4.3
cp178-177	0	1.0
cp179-178	0	0
**Loop 9–10 region and flanking sites**		
cp188-187	0	0
cp189-188	0	0.7
cp190-189	0.4	36.3
cp191-190	0.3	19.8
cp192-191	0	11.0
cp193-192^a^	0.2	17.2
cp194-193	1.0	39
cp195-194	0	3.3
cp196-195	0.2	3.1
cp197-196	0.3	3.8
cp198-197	0.1	3.8
cp199-198	0	0
**Published cp-mCherry** c		
cp184	<0.5	18
cp193	<0.5	1
cp193g7	69	100

* Circular-permutated mCherry variants were numbered by the primary amino acid sequence of mCherry as shown in **Fig. 1C**. Variants are labeled with the new amino and carboxy termini (e.g. cp159-158 has the new N-terminus from the native carboxy amino acids 159 to 236 and the new C-terminus from the amino residues 1 to158).

a Fluorescent variants from the initial screening in each region.

b Aligns to cp-EGFP in GCaMP2.

c Numbered by the amino acid sequence of DsRed as shown in **Fig. 1C** [18], [19]. cp184-mCherry corresponds to cp191-190, with three duplicated residues at N-terminus (188-190). The cp193-mCherry and cp193g7 (with 6 mutated amino acids) correspond to cp197-196, with three duplicated residues (197-199) at the C-terminus.

**Table 2:** Properties of circular-permutated mCherry variants.

Protein^a^	Extinction coefficient^b^ (M^-1^ cm^−1^)	Quantum yield^c^	Relative brightness^d^ (% of mCherry)	Brightness of protein^e^ (% of mCherry)
mCherry	91,000^f^	0.22	100	100
cp159-158	91,000	0.21	95	37.4
cp160-159	89,000	0.21	93	22.0
cp175-174	91,000	0.20	91	41.5
cp190-189	88,000	0.21	92	59.1
cp191-190	93,000	0.20	93	53.3
cp193-192	93,000	0.20	93	51.7
cp194-193	90,000	0.20	90	60.6
cp184	26,600^g^	0.22	ND^i^	37.0
cp193g7	42,000^g^	0.23	ND^i^	60.6

a All circular-permutated variants from native mCherry sequence shown in this table, except cp184 (not determined), have the same excitation (587 nm) and emission (610 nm) maxima as mCherry. The excitation and emission maxima of the cp193g7, with 6 amino acid mutations, are 580 nm and 602 nm, respectively [19].

b Extinction coefficients were measured by alkali-denatured chromophore method.

c Quantum yields were measured using mCherry as the reference standard.

d Relative brightness of chromophore (extinction coefficient × quantum yield) was compared with mCherry (91,000×0.22).

e Fluorescence of cp-mCherry relative to mCherry with fixed protein concentration (BCA assay).

f Our data; the published data are 72,000 [7], 78,000 [9].

g Published values [18], [19], which were based on the protein quantification (absorption at 280 nm).

i Not determined.

### Circular Permutation of mKate

**Figure 3:**
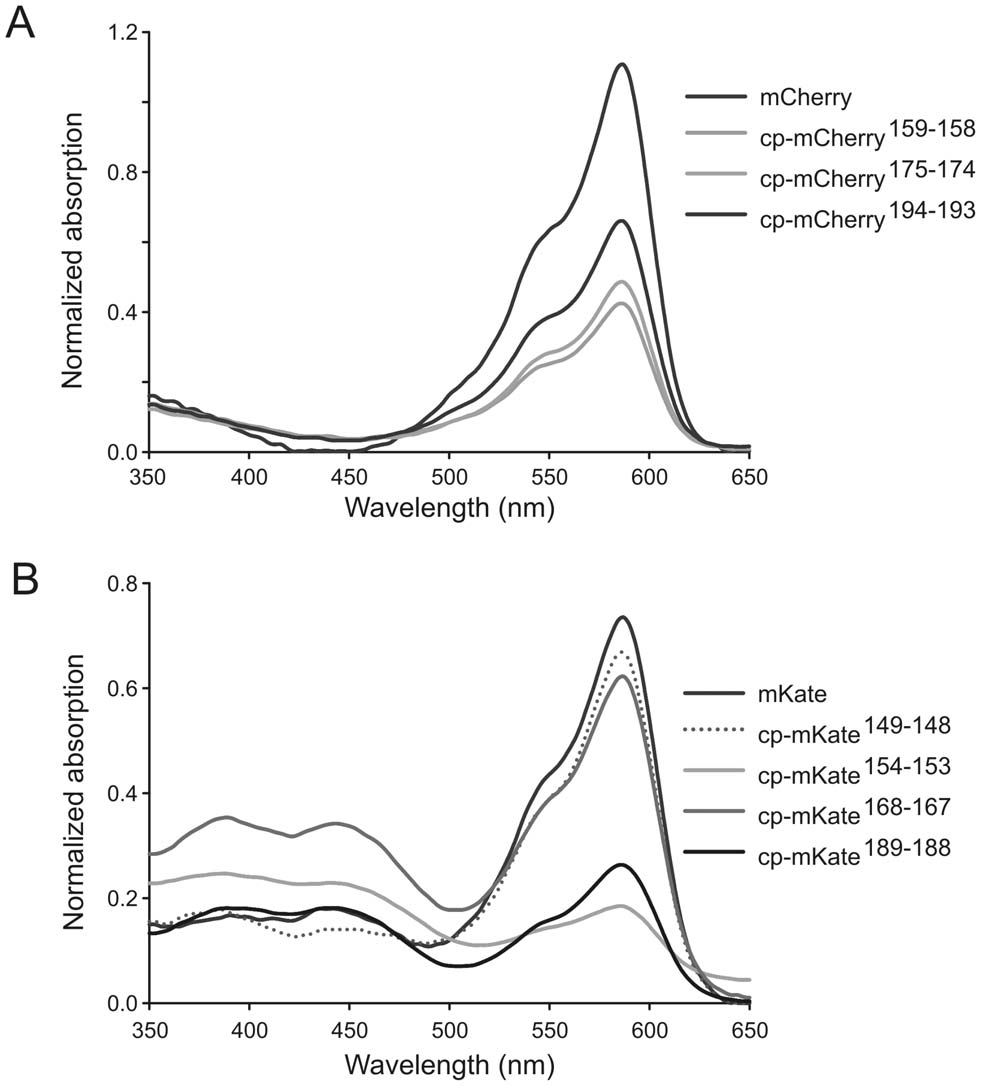
Absorption spectra of cp-mCherry and cp-mKate variants. Spectra were normalized to the 280 nm absorption for each protein. **A.** Absorption spectra of mCherry and cp-mCherry variants. **B.** Absorption spectra of mKate and cp-mKate variants.

We performed a similar circular permutation analysis of the monomeric far-red protein mKate, which derives from the sea anemone *Entacmaea quadricolor* and shares structural similarities with mCherry. Based on structural and sequence similarities, we first tested three cp-mKate variants that corresponded to the brightest cp-mCherry variants in each region of permissive sites. Fluorescence was detected in colonies transformed with cp-mKate^151-150^, cp-mKate^167-166^, and cp-mKate^189-188^. As with the cp-mCherry evaluation, we also screened the sequences flanking the three identified circular permutation sites in mKate for additional tolerant sites. In contrast to the slow development of fluorescence in cp-mCherry colonies, most of the fluorescent cp-mKate variants exhibited appreciable fluorescence after 24 h incubation at 37°C (**Table 3**). cp-mKate^189-188^ is the brightest circular permutation variant in *E.coli*. However, analysis of purified proteins in 20 mM HEPES (pH 7.4) indicated that the brightness of cp-mKate^149-148^, mKate^151-150^, cp-mKate^167-166^, and cp-mKate^168-167^ was quite high, exceeding 80% of fluorescence of native mKate, indicating highly efficient fluorescent configurations. The 442 nm absorption peak (**Fig. 3B**), which emits green fluorescence at 532 nm, revealed a slight augmentation of green fluorescence in cp-mKates. The ratio of absorption at 588 nm and 442 nm (**Table 4**) indicated a relatively high percentage of green components in cp-mKate^154-153^, cp-mKate^168-167^, cp-mKate^187-186^, and cp-mKate^189-188^, whereas cp-mKate^149-148^ displays a fluorescence spectrum similar to the native mKate. The quantum yield at 588 nm for the cp-mKates is only slightly lower than mKate, whereas the ratio of A_588_/A_280_ indicated that a number of cp-mKate molecules (cp-mKate^154-153^, cp-mKate^187-186^, cp-mKate^189-188^) have a substantially lower absorption at 588 nm with matched protein concentration (**Table 4**).

**Table 3:** Screening of circular-permutated mKate variants in *E.coli*.

Variant *	Colony brightness 24 h at 37°C (% of mKate)	Colony brightness 72 h at 2–8°C (% of mKate)
mKate	100	100
pRSET-A	0	0
cp139-138^a^	0	0
cp140-139^a^	0	0
cp141-140^a^	0	0
cp144-143^a^	0	0
**Loop 7-8 region and flanking sites**		
cp148-147	0	0
cp149-148	1.1	32.3
cp150-149	0.2	11.7
cp151-150^b^	7.5	28.4
cp152-151	5.1	21.4
cp153-152	2.9	20.8
cp154-153	10.9	19.4
cp155-154	0	0
cp156-155	0	0
**Loop 8–9 region and flanking sites**		
cp164-163	0	0
cp165-164	1.9	13.2
cp166-165	10.2	24.7
cp167-166^c^	6.6	21.6
cp168-167	3.6	22.2
cp169-168	1.6	16.3
cp170-169	0	0
**Loop 9–10 region and flanking sites**		
cp180-179	0	0
cp181-180	0	0
cp182-181	11.5	12.6
cp183-182	23.7	30.0
cp184-183	18.7	28.4
cp185-184	9.7	17.5
cp186-185	21.2	27.7
cp187-186	26.7	30.8
cp188-187	9.9	19.7
cp189-188^d^	33.9	55.2
cp190-189	12.1	18.3
cp191-190	8.3	15
cp192-191	0.4	6
cp193-192	0	0
cp194-193	0	0

* The circularly permuted variants are labeled with the new amino and carboxy termini.

a Align to cp-EGFP in GCaMP2.

b Aligns to cp-mCherry^159-158^.

c Aligns to cp-mCherry ^175-174^.

d Aligns to cp-mCherry^194-193^.

**Table 4:** Properties of circular-permutated mKate variants.

Protein^a^	Absorption^b^ (A_588_/A_442_)	Quantum yield^c^ (588 nm)	Absorption^d^ (A_588_/A_280_)	Brightness of protein^e^ (% of mKate)
mKate	4.13	0.33	0.74	100
cp149-148	4.79	0.33	0.68	90.1
cp151-150	3.25	0.31	0.61	83.6
cp154-153	0.81	0.28	0.19	21.2
cp166-165	2.88	0.28	0.58	70.5
cp167-166	2.73	0.32	0.61	81.6
cp168-167	1.8	0.30	0.65	84.0
cp187-186	1.45	0.30	0.25	30.4
cp189-188	1.47	0.29	0.27	36.3

a Red fluorescence of all circular-permutated mKate variants shown in this table, with a N-terminal leader peptide from pRSET vector, have the same excitation (588 nm) and emission (620 nm) maxima as mKate; whereas the published emission maximum of mKate are 635 nm [9], and 625 nm [24].

b Absorption maxima for red and green chromophores are 588 nm and 442 nm, respectively.

c Red fluorescence quantum yield at 588 nm excitation were measured using mKate as the reference standard.

d Ratio of red chromophore absorption at 588 nm and protein absorption at 280 nm.

e Fluorescence of cp-mKate relative to mKate with fixed protein concentration (BCA assay).

We also screened cp-mKate variants that correspond to the site in cp-EGFP used to develop the GCaMP family of calcium sensors [2], [3], based on structural and sequence alignments of mKate and EGFP. Colonies transformed with cp-mKate^139-138^, cp-mKate^140-139^, cp-mKate^141-140^, cp-mKate^144-143^ did not display red fluorescence, even when incubated up to 7 days at 2–8°C (**Table 3**).

### Truncations of cp-mKate

To explore the possibility that the fluorescence of individual circular permutations is influenced by N- or C-terminal residues, we next determined the sensitivity of fluorescence to deletions at the amino and carboxy termini of the fluorescent cp-mKate permutants. A series of variants with truncated amino or carboxy ends were constructed. In this regard an individual construct could be regarded as either an amino or carboxy deletion; thus cp-mKate^154-150^, for example, is an amino truncation of cp-mKate^151-150^, or a carboxy truncation of cp-mKate^154-153^. As shown in **Table 3**, for Loop 9–10 region of cp-mKates, the fluorescent variants contain the N-terminal residue from 182 to192 and the C-terminal residue from 181 to 191, respectively). Most of the truncated variants from cp-mKate^183-182^ and cp-mKate^182-181^ markedly decreased fluorescence in *E.coli*, except the cp-mKate^185-182^, which had similar performance to that of the cp-mKate^183-182^ (**Table 5**). The N- and C-terminal boundary for truncation in which fluorescence was preserved were 192 and 181, respectively, indicating that truncations within the region of circular permutation were tolerated. Similar experiments were performed in the region of Loop 7–8 and Loop 8–9 cp-mKate variants, indicating that the amino and carboxy boundaries for truncation were residues 154 and 148 for Loop 7–8 region, and 169 and 164 for Loop 8–9 region (data not shown).

**Table 5:** Truncation of circular-permutated mKate variants.

Variant	Colony brightness 24 h at 37°C (% of mKate)	Colony brightness 72 h at 2–8°C (% of mKate)
cp183-182*	21.4	24.6
cp185-182	23.1	24.2
cp188-182	15.1	13.3
cp191-182	7.9	11.5
cp193-182	0	0
cp182-181*	16.9	16.9
cp186-181	2.6	2.2
cp191-181	1.8	3.1
cp192-181	0.2	1
cp193-181	0	0
cp181-180*	0	0
pRSET-A	0	0
mKate	100	100

*Original symmetric cp-mKate.

### Crystal Structure of cp-mKate

**Figure 4:**
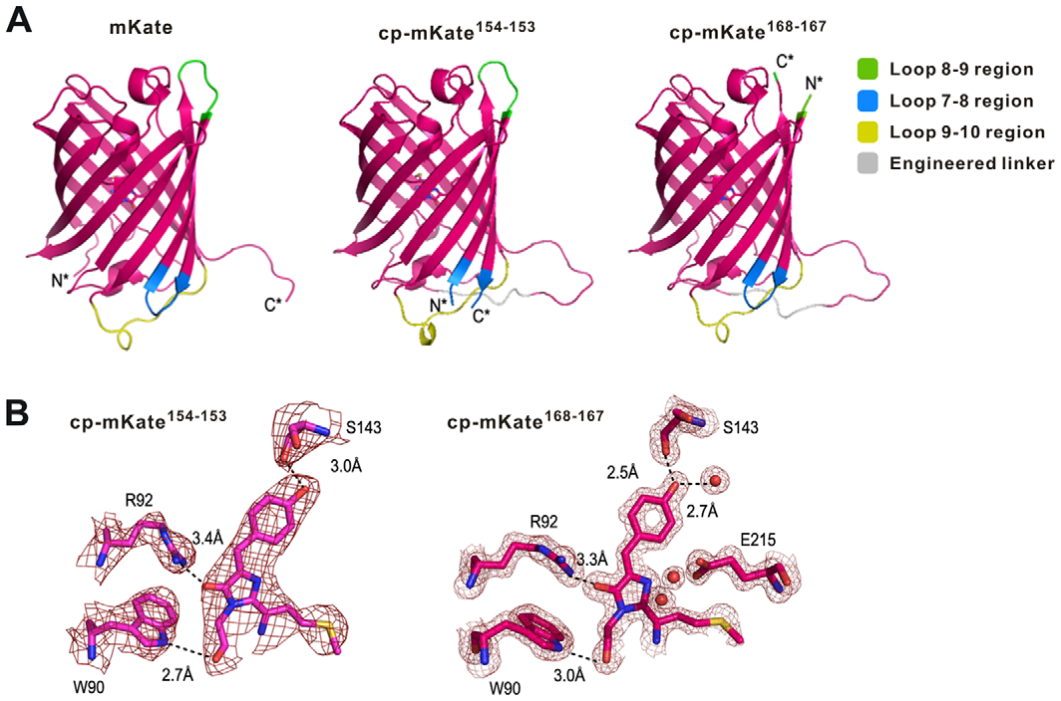
Crystal structure of cp-mKate^154-153^ and cp-mKate^168-167^. **A.** Cartoon presentation of mKate (pH 7.0), cp-mKate^154-153^ and cp-mKate^168-167^. The figures are drawn with PyMol (DeLano Scientific). **B.** 2mFo-Fc electron density map near chromophore region. The map is contoured at 1.0σ.

X-ray crystallography on cp-mKate^154-153^ and cp-mKate^168-167^ allowed us to resolve the structure of the two permutants at 3.0Å and 1.7Å resolution, respectively (**Table 6** and **Fig. 4**). The resolved structure is quite similar to that previously reported for wild-type mKate [13] (route mean square deviations of 0.31Å and 0.23Å, respectively), revealing an elliptical β-barrel that is properly folded in cp-mKate^168-167^ and indistinguishable in tertiary structure to that of wild-type mKate (**Fig. 4A**). The linker that connects the two reoriented components of the circularly permutated molecule appears in the loop structure and locates at one end of the β-barrel (**Fig. 4A**). Minor conformational changes in cp-mKate were restricted to the loop structures at both ends of the β-barrel and may account for the reported red–green shift. The chromophore resides entirely in *cis*-conformation, however, with hydrogen-bonding with Trp 90, Arg92 and Ser143 (**Fig. 4B**).

**Table 6:** X-ray Data Colletion and Refinement Statistics of cp-mKate^154-153^ and cp-mKate^168-167^.

	cp-mKate^154-153^	cp-mKate^168-167^
**Data collection**		
X-ray source	CHESS A1	CHESS A1
Wavelength (Å)	0.978	0.978
Space group	P2_1_2_1_2_1_	C2
**Unit cell parameters**		
a, b, c (Å)	71.46, 71.44, 367.73	109.357, 93.280,194.370
α, β, γ (°)	90.0, 90.0, 90.0	90.0, 96.52, 90.0
Resolution (Å)	38.98-3.00 (3.11-3.00)	38.31-1.75 (1.81-1.75)
**No. of reflections**		
Total	330773 (32752)	180131 (15380)
Unique	37589 (3680)	45032 (4660)
Completeness (%)	99.9 (100.0)	92.5 (79.6)
Redundancy	8.8 (8.9)	4.0 (3.3)
* I*/σ(*I*)	15.3 (2.0)	39.4 (7.26)
* R*_meas_ (%)	11.9 (47.7)	5.1 (14.5)
**Refinement**		
*R_work_*/*R_free_* (%)	21.7/27.8	18.0/21.1
**r.m.s. deviations**		
Bond length (Å)	0.009	0.007
Bond angles (°)	1.590	1.164
**No. of atoms**		
Protein	14974	16094
Water	2	1453

## Discussion

The generation of large numbers of circular permutations by tandem template PCR and fluorescence screening of bacterial colonies is a highly efficient approach to exploring the potential permutation variants of fluorescence proteins. We have systematically examined mCherry and mKate by creating circular permutants at the loop regions in which the greatest flexibility would be expected. In attempting to develop a highly fluorescent circularly permutated red protein, design strategies aimed at structurally mimicking cp-EGFP, which is the basis for the successful GCaMP Ca^2+^ sensors [3], [20], [21], proved not to be a successful approach, as the analogous cp variants of both mCherry and mKate failed to show significant fluorescence. Structural analysis revealed three highly homologous loop regions in mCherry and mKate, and these areas along with flanking sites were systematically explored. Each region tolerated circular permutation, but fluorescence varied markedly, with most efficient permutation sites tending to occur within the central loop regions.

As shown in **Table 1** and **Table 2**, the brightest mCherry variant (cp-mCherry^194-193^) retained 60.6% of the brightness of native mCherry on a protein basis, but strong fluorescence was also observed for circular permutations cp-Cherry^159-158^, cp-Cherry^160-159^, cp-mCherry^175-174^, cp-mCherry^190-189^, cp-mCherry^191-190^ and cp-mCherry^193-192^. All of the cp-mCherry variants displayed similar absorption spectra as native mCherry (**Fig. 3A**). Moreover, the red chromophore in cp-mCherry variants and mCherry are functionally similar, having nearly equivalent extinction coefficient and quantum yield values. Thus, the marked difference in relative brightness between cp-mCherry variants and native mCherry when evaluated on a chromophore and equivalent concentration of protein basis indicates that the decreased brightness of the cp-mCherry proteins is due to incomplete folding of a significant fraction of the protein. This interpretation is further supported by the fact that the 6 amino acid mutations in cp193g7, which have been shown to improve protein folding efficiency, resulted in a much higher fluorescence than observed in cp193-mCherry [19], similar to the brightness effect of the superfolder green fluorescent protein [22].

In general the fluorescence of cp-mCherry permutants from native mCherry developed more slowly, and achieved much higher brightness when bacteria were further incubated at lower temperature. By contrast, cp-mKate variants developed significant fluorescence in bacteria by 24 h at 37°C (**Table 3**). The brightest variant in the bacterial assay was cp-mKate^189-188^, with colonies achieving 55.2% of the brightness of native mKate after 72 h incubation. However, the brightness of bacterial colonies did not strictly correlate with protein fluorescence, and proteins isolated from only modestly fluorescent colonies were among the brightest proteins identified. Thus although cp-mKate^189-188^ demonstrated the brightest colony fluorescence, the purified protein displayed 36.3% of native mKate fluorescence, whereas the relative fluorescence of cp-mKate^149-148^, cp-mKate^151-150^, cp-mKate^167-166^, and cp-mKate^168-167^ exceeded 80% at pH 7.4. The higher fluorescence of these proteins may relate to cytosolic factors such as pH, resulting in higher percentage of properly folded protein and mature red chromophore in the purified proteins.

Individual sites for the modification of mKate that tolerate circular permutation or splitting have been previously reported. A voltage probe used a cp-mKate(180) [23] that contained 3 duplicated amino acids at the N-ternimus and otherwise corresponded to the moderately bright cp-mKate^183-182^. A reported functional split site for mKate (151) for a BIFC system [24] belongs to the highly fluorescent Loop 7–8 region (**Table 3**). We also confirmed the tolerance of selected sites to peptide insertions, which was found to be robust (data not shown).

Although mKate and its variants have been widely used as red fluorescent proteins, these proteins exhibit green fluorescence to a variable degree [25]. We found that circular permutation of mKate enhanced green fluorescence in several constructs, augmenting absorption at 442 nm. As any green fluorescence of mKate proteins will contribute to the total fluorescence after alkaline-denaturation, a precise measurement of the extinction coefficient of the red chromophore in cp-mKates by this method is not valid. As with mCherry circular permutants, red fluorescence quantum yield is only slightly decreased in cp-mKates. In the case of mCherry, the extinction coefficients of circularly permutated and wild-type proteins are quite similar, and the decrease in the A_587_/A_280_ absorption ratio indicates that the contribution of poorly folded proteins is the major factor in loss of brightness (**Table 2** and **Fig. 3A**). However, for mKate variants with lower brightness than the native protein, similar A_588_/A_280_ ratios between these forms reflects not only the effect of improperly folded protein, but the extent to which permutation has resulted in a red–green shift. Interestingly, we found very bright cp-mKates with substantial red–green shifts, such as cp-mKate^168-167^, and very bright variants without a substantial shift, such as cp-mKate^149-148^ (**Table 4** and **Fig. 3B**). The overall brightness of these constructs suggests minimal effects of incomplete folding at pH 7.4, whereas loss of brightness in variants with similar red–green shifts, such as seen in a comparison of cp-mKate^168-167^ and cp-mKate^187-186^, indicates less efficient folding and chromophore stabilization in the latter variant (**Table 4** **and** **Fig. 3B**). cp-mKate^149-148^ was the brightest protein identified with over 90 percent of native brightness, but 3 additional variants (cp-mKate^151-150^, cp-mKate^167-166^, and cp-mKate^168-167^) exceed 80% of mKate fluorescence, constituting new permutants available for implementation as sensor or complementation pairs. Moreover, the discovery of bright sensors with substantial green fluorescence, such as cp-mKate^168-167^, or a stabilized cp-mKate^154-153^, may be useful in the development of green/red ratiometric sensors.

Truncation variants showed that in each region of cp-mKate, there are minimum native N-terminal and C-terminal fragments required to maintain fluorescence. For example, the minimum C-terminal and N-terminal fragments were 192-231 and 1-181, for Loop 9–10 region cp-mKate. The ability to truncate variants without loss of significant fluorescence provides significant flexibility in the linkage of these permutants to other functional peptides.

Finally, crystallization and structural analysis of cp-mKate^154-153^ and cp-mKate^168-167^ revealed the expected tertiary structure previously reported for mKate [16], with only slight variations around the permutation point. Future studies will be directed at determining the structural basis for fluorescence variation in circular permutants of mKate.

In summary, we report several highly fluorescent circularly permutated variants of mCherry and mKate. These variants are grouped in 3 regions and constitute the brightest red circularly permutated proteins with native protein sequences reported to date. The reported bright circularly permutated mKate proteins, and further stabilized mCherry variants, should provide additional candidates for the construction of red sensors and complementation tools.

### Accession Numbers

Atomic coordinates and structure factors have been deposited in the RCSB Protein Data Bank under ID code 3rwt and 3rwa.
